# Manaaki – a cognitive behavioral therapy mobile health app to support people experiencing gambling problems: a randomized control trial protocol

**DOI:** 10.1186/s12889-020-8304-x

**Published:** 2020-02-06

**Authors:** Gayl Humphrey, Joanna Chu, Nicki Dowling, Simone Rodda, Stephanie Merkouris, Varsha Parag, David Newcombe, Elsie Ho, Vili Nosa, Rebecca Ruwhui-Collins, Robyn Whittaker, Chris Bullen

**Affiliations:** 1 National Institute for Health Innovation, University of Auckland, Private Bag 92019, Auckland, New Zealand; 2 Center for Addiction Research, University of Auckland, Private Bag 92019, Auckland, New Zealand; 3 School of Psychology, Deakin University, Geelong, Victoria Australia; 4 Melbourne Graduate School of Education, University of Melbourne, Melbourne, Victoria Australia; 5 Social and Community Health, University of Auckland, Auckland, New Zealand; 6 Pacific Health, University of Auckland, Auckland, New Zealand; 7 Hapai te Hauora, Auckland, New Zealand; 8 Waitemata District Health Board, Auckland, New Zealand

**Keywords:** Problem gambling, mHealth, CBT, App, Smartphone, Self-directed, Behavior change, App Utilization

## Abstract

**Background:**

The low utilisation of current treatment services by people with gambling problems highlights the need to explore new modalities of delivering treatment interventions. This protocol presents the design of a pragmatic randomized control trial aimed at assessing the effectiveness and acceptability of cognitive behavioral therapy (CBT) delivered via a mobile app for people with self-reported gambling problems.

**Methods:**

An innovative CBT mobile app, based on Deakin University’s GamblingLess online program, has been adapted with end-users (Manaaki). Six intervention modules have been created. These are interwoven with visual themes to represent a journey of recovery and include attributes such as avatars, videos, and animations to support end-user engagement. An audio facility is used throughout the app to cater for different learning styles. Personalizing the app has been accomplished by using greetings in the participant’s language and their name (e.g. Kia ora Tāne) and by creating personalized feedback.

A pragmatic, randomized control two-arm single-blind trial, will be conducted in New Zealand. We aim to recruit 284 individuals. Eligible participants are ≥18 years old, seeking help for their gambling, have access to a smartphone capable of downloading an app, able to understand the English language and are willing to provide follow-up information at scheduled time points. Allocation is 1:1, stratified by ethnicity, gender, and gambling symptom severity based on the Gambling Symptom Assessment Scale (G-SAS). The intervention group will receive the full mobile cognitive behavioural programme and the waitlist group will receive a simple app that counts down the time left before they have access to the full app and the links to the data collection tools. Data collection for both groups are: baseline, 4-, 8-, and 12-weeks post-randomisation. The primary outcome is a change in G-SAS scores. Secondary measures include changes in gambling urges, frequency, expenditure, and readiness to change. Indices of app engagement, utilisation and acceptability will be collected throughout the delivery of the intervention.

**Discussion:**

If effective, this study will contribute to the improvement of health outcomes for people experiencing gambling problems and have great potential to reach population groups who do not readily engage with current treatment services.

**Ethics approval:**

NZ Health and Disability Ethics Committee (Ref: 19/STH/204)

**Trial registration:**

Australian New Zealand Clinical Trial Registry (ANZCTRN 12619001605189) Registered 1 November 2019.

## Background

Internationally, standardised prevalence rates of problem gambling range from 0.5 to 7.6%, with an average rate across all countries of 2.3% [1]. In New Zealand, the prevalence of people with some level of risk for gambling problems is estimated at 6.8% of the population, whereby approximately 0.3% of the population experience problem gambling with a further 1.5% classified with moderate-risk gambling and 5.0% with low-risk gambling [2]. Māori (indigenous people in New Zealand) and Pacific peoples have higher rates of all levels of gambling risk and have more persistent gambling problems over time [3, 4]. The negative sequelae of gambling problems can include financial harm, relationship dysfunction and conflict, emotional distress, health decrements, cultural harm, reduced work or study performance, and criminal activity [5].

### Treatment approaches to problem gambling

Traditionally, individuals seeking help for gambling-related issues have obtained treatment from general physicians or specialised counselling services through face-to-face sessions. Treatment approaches that use cognitive behavioural therapy (CBT) appear to help reduce gambling activity and related behaviours in the short term [6]. Motivational interviewing (MI) strategies also provide benefit by reducing aspects of gambling behaviour, such as cutting back or reducing the amount of money gambled [7, 8]. Nevertheless, fewer than 10% of people with a gambling problem are in face-to-face treatment at any one time [2, 9], with most seeking treatment only in response to a significant life crisis [10]. Reasons and barriers for the lack of face-to-face uptake include cost, geographical distance, transport limitations, conflicting commitments, fears of stigmatisation and shame, and privacy concerns [11, 12]. New approaches are therefore needed to enhance access to address gambling-related harm.

Internet-delivered interventions have grown over the past decade and respond to many of the barriers reported as being responsible for the low uptake of face-to-face services [13, 14]. Nonetheless, few studies have evaluated self-directed gambling interventions delivered over the internet. A recent systematic review revealed that the two available high-intensity, self-directed, structured, online gambling interventions [15, 16] were as effective on all outcomes following treatment compared to those obtained from face-to-face treatments [17]. Since then, an online self-directed CBT program (GamblingLess) has been developed by some of the researchers for the proposed trial. The program was developed as a comprehensive and intense intervention that emulates the intensity and depth of a face-to-face cognitive-behavioural intervention, and from which briefer and more targeted online and app-based self-directed interventions can be developed. A recent pragmatic randomised trial in Australia examined the programme delivered without any practitioner (clinician) guidance or delivered with practitioner guidance at 8-weeks, 12-weeks, and 24 months from the pre-intervention assessment (Dowling N, Merkouris S, Rodda S, Smith D, Lavis T, Lubman D, et al. GamblingLess: For Life: A pragmatic randomised trial of an online cognitive-behavioural program for disordered gambling. *in preparation*, [18–20]). They found statistically significant improvements in gambling symptom severity, gambling urges, gambling frequency, gambling expenditure, and psychological distress within both treatment groups across the evaluation period. There were also significant improvements in quality of life for the guided self-directed group. At the end of the 24-month evaluation period, 69% of the sample were recovered or improved on gambling symptom severity. The programme was also positively evaluated by both end-users and guides. However, further controlled studies are needed to conclusively confirm the efficacy of this intervention.

### Mobile health (mHealth): a new opportunity for problem gambling

The technological capability of smartphones is opening up new ways to consider providing intervention and relapse prevention support [21]. Mobile health (mHealth) interventions, which include texting, self- directed activity and sensors within (e.g. step counter) or tethered to, mobile phones (smartwatch) to address health issues across the continuum, have great potential for public health impact because of their broad reach and convenience [22].

Smartphone apps have been shown to support self-management and behaviour change for smoking cessation [23], cardiac rehabilitation [24], healthy lifestyle [25], diabetes [26], HIV [27], nutrition [28], mental illness [29] and youth driving [30]. In a recent meta-analysis of smartphone interventions for mental health problems, smartphone apps outperformed waitlist controls with small to medium effect sizes [31]. The effect size was greater for apps that had a theoretical basis in CBT. Apps that were compared with active control were not found to be significantly different.

The use of mobile apps remains largely untapped in the gambling domain [32]. Our previous work, SPGeTTI, an application that utilised innovative technology including GPS and notifications to support gambling relapse prevention reported positive participant engagement during the formative development phase [33]. Despite technical and recruitment challenges, participants in the study reported an ongoing interest in having smartphone apps as potential tools to support them to quit or reduce their harmful gambling [34]. Other mHealth studies have also reported similar positive engagement and acceptability with smartphone apps [35, 36].

Concurrent with the growth in mHealth tools is the exponential growth in smartphone ownership internationally and in New Zealand [37]. A Pew Research Report also highlights that the age gap in smartphone ownership is also narrowing [38]. Globally, the use of the advanced features available on mobile phones surpasses the use of basic features such as text messaging [39]. In New Zealand, a 2014 survey reported that smartphone ownership was more common among Māori and Pacific people (70%) than Europeans (55%) [40]. In addition, 92% of New Zealand households have access to a mobile phone [41], with no differences in internet access or smartphone ownership by ethnicity or education, or age [41]. Thus, there is considerable potential in New Zealand to leverage mHealth technology in addressing gambling-related problems.

## Rationale for research

The low utilisation of current treatment services by people with gambling problems highlights the need to explore new modalities of delivering treatment interventions to reach those groups who prefer to self-manage and may prefer not to access (or remain in) face-to-face services. The positive results from internet-delivered gambling intervention programmes are important, as is the emerging evidence that interventions delivered using mobile phones have the potential to reach a wide group of people experiencing gambling problems. The early gambling research into mHealth indicates that these modalities are acceptable and feasible. The combination of high smartphone ownership and demand for health apps provide both the opportunity and vehicle to reach a significant portion of the population who may not readily engage with current health or treatment services.

## Objective

The primary aim of the study is to evaluate the effectiveness of a smartphone application intervention for people with self-reported gambling problems. Specifically, we hypothesize that compared with a wait-list control, the use of a self-directed and personalized CBT based app [Manaaki] for 12 weeks post-randomization will lead to:

1. Reduction in gambling symptom severity (primary outcome)
2. Reduction in gambling urges, gambling frequency, gambling time and gambling expenditure and improved readiness, willingness, and ability to change (secondary outcomes)

A secondary aim is to explore app engagement, utilization and acceptability.

## Methods

### Design

This study is a pragmatic, randomised, wait-list controlled, single-blinded, two-arm trial. Eligible individuals will be randomly allocated to the Manaaki app (intervention group) or the waitlist control group. Data will be obtained from all participants at baseline, then at 4-, 8-, and 12-weeks post-randomisation. The study has been approved by the New Zealand Health and Disability Ethics Committee (Ref 19STH204) and any changes will be reported as per ethics standard operating procedures and policies. The study is also registered with the Australian New Zealand Clinical Trial Registry (ACTRN12619001605189p), and the protocol conforms to the SPIRIT statement [42].

### Participants

A total of 284 individuals residing in New Zealand will be recruited. Individuals will be eligible for inclusion in the study if they are aged 18 years and over, have an interest in seeking help for their own gambling, have access to a smartphone capable of downloading an app, have access to the internet, possess adequate knowledge of the English language and are willing to provide follow-up information at scheduled time points. Individuals who indicate that they do not meet the inclusion criteria will be ineligible and unable to complete the process to activate the app. As this is a pragmatic study, accessing other help or treatment services is not an exclusion criterion.

### Data safety monitoring and auditing

This study does not meet the requirements for a data safety monitoring committee and as such one has not been established. The study will be audited prior to recruitment initiated.

### Setting

This study will be conducted nationwide in New Zealand. Methods to recruit individuals will include media advertising, peer referral and promotion through appropriate networks, relevant agencies, community groups and peer networks. Media advertising channels will include print media and online advertisements (including websites such as TradeMe, Facebook and Google Adwords) and radio. Recruitment will also be promoted via links to health-specific websites. Networks through our study partners, Hāpai Te Hauora, who facilitate the national coordination service for minimising gambling harm, will also be utilised.

### Study procedures

**Fig. 1:**
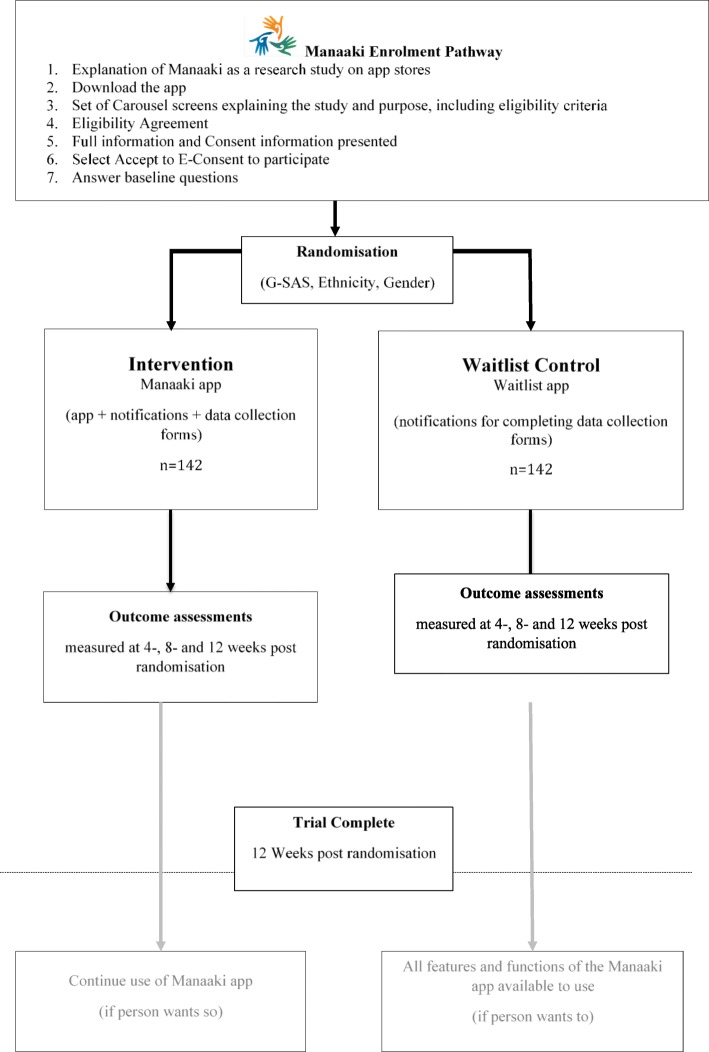
Manaaki Enrolment Pathway

Figure 1 shows the study procedure. Interested participants will download the app from either the App Store or Google Play. The study participant information will be presented to them. If they have questions, the study contact details are included in the information section of the app, and participants have an opportunity to talk and ask questions with a researcher about the study before deciding to participate. Participants who wish to participate will be presented with a summary of what participation means (i.e. what they are agreeing to) and they can either provide their electronic consent (e-consent) or decline. If a person does not wish to participate, they can select - decline. A thank you for considering participating in the Manaaki study will be displayed. Validation criteria will be utilised where practicably possible; for example, downloading can only be completed by individuals within NZ by placing a location boundary restriction on the app in the App stores, and if month and year of birth entered indicates that the participant is under 18 years, then a “thank you but you are not eligible” pop-up message will appear. Consent to collect app use data from all participants after their 12-week study period is complete will also be sought.

Following e-consent, participants will be guided through the baseline data collection questions, which include the criteria for randomisation (ethnicity, sex and G-SAS 0–30 or 31–48). Once all baseline questions completed, participants will be randomised to the Manaaki intervention app or the waitlist control app. To prevent participants who are randomised to the Waitlist control app from uninstalling and reinstalling the app in a desire to receive the Manaaki intervention app, the unique phone ID will be used to confirm an existing login. The waitlist participants will be able to access the full app at the completion of the 12-week study period.

All data collection assessments (baseline, 4, 8 and 12-weeks) will be embedded within the app. A koha (gift) of NZ$60 will be provided to each participant in the form of a grocery voucher. The koha will be divided into three equal amounts of NZ$20 and administered at the completion of each follow-up data point.

All data is housed on a password protected secure server. Access to the final data set will be to the study statistician and appropriately approved study researchers.

### Randomization

Upon completion of the baseline data collection, participants will be randomised in a 1:1 ratio to one of the two groups. Stratified block randomisation (created by the study statistician), using block sizes of 2 and 4, will be used to randomly allocate participants. To ensure balance on potential confounders, randomisation will be stratified by ethnicity (Māori, Pacific, Other), sex and gambling symptom severity using the Gambling Symptom Assessment Scale (G-SAS): dichotomised to mild to moderate gambling symptom severity (G-SAS score 0–30) and severe to extreme gambling symptom severity (G-SAS score 31–40) to ensure a balance in these key characteristics.

The randomisation process will be managed within a secure backend server. Upon completion of the baseline questions and the receipt of these in the server, the randomisation protocol will be activated. When the participant clicks “next” the outcome of the randomisation will make available either the Manaaki app or Waitlist control app.

### Blinding

The trial will be single-blinded as participants will be aware of the group to which they have been allocated. All members of the research team will be blinded to treatment allocation.

### Study intervention

#### Manaaki app

Participants randomized into the intervention group will have full access to the Manaaki app. The content of the app was adapted from GamblingLess, an online CBT program evaluated in an Australian pragmatic trial (Dowling N, Merkouris S, Rodda S, Smith D, Lavis T, Lubman D, et al. GamblingLess: For Life: A pragmatic randomised trial of an online cognitive-behavioural program for disordered gambling. *in preparation*, [19]). The program incorporates content related to motivational enhancement, cognitive and behavioural strategies, and relapse prevention strategies. Formative work has been conducted to enhance the tailoring of content and develop content that was deemed culturally relevant and appropriate for New Zealand users. The program was also redeveloped as an interactive application that can be used on a mobile phone. Table 1 details the modules, conceptual framework, and intervention elements of the Manaaki app.

**Table 1:** Domains and intervention elements for the programme

Modules	Conceptual Framework	Underpinning Intent	Key Intervention Elements
Knowing Myself (and my gambling)	Developing self-awareness and insight [44]	Designed to provide personalised feedback, goal setting, and understanding of gambling motivations, triggers, and/or consequences	Personalised feedback on gambling symptom severity and gambling behaviour Reflecting on their goal of quitting or cutting back reasons for gambling My gambling triggers My negative gambling consequences My reasons for gambling
Getting Ready (to make changes)	Targeting thoughts and feelings and activating behaviours [46]	Designed to enhance readiness and confidence to gamble less, helping to shape thoughts and values to help make change	Am I ready to gamble less? Knowing my values Knowing my strengths The benefits of gambling less My confidence Deciding on my goal for change
Taking Control (right now)	Targeting practical behaviours and identification of situational and contextual triggers	Designed to identify strategies that can be used to “contain” the gambling in the short-term, directs to other useful tools such as venue exclusions	My previous strategies to gambles less Limiting access to venues Limiting access to money Guidelines for gambling safely Resisting social pressures
Taking Actions (that last)	Activating personal strengths and resources and enhancing belief for successful change [47, 48]	Designed to identify strategies and skills that can be used to ensure longer-term success in gambling less	My budget My enjoyable activities Learning to relax The tricks that keep me gambling My gambling thinking traps Gamblers fallacy, Chasing, Illusion of control, Near misses
Managing Urges (to cope with real situations)	Reframing thoughts and reflecting on your future self [47, 48]	Designed to cope with gambling urges and cravings	Previous attempts to manage my gambling urges The three ‘Ds’: delay, distract and discuss My brief relaxation strategies My brief imagery strategies How I rationalise my gambling Urge surfing My urge management reminders
Change for GOOD (and building a new future)	Relapse prevention [49]	Designed to prevent gambling relapse in the future	Identifying my high-risk situation and my thoughts and feelings My seemingly irrelevant decisions My willpower breakdown My decision consequences Learning from my lapses

Participants are presented with a range of themes and options to help them reflect on what they hope to achieve from the program, as well as being supported via various modules and topics. The program is designed to be used in a non-linear way, and participants can navigate around the various modules and submodules in any order and complete all or any activities within each. The completed modules and activities are visible on the main navigation screen.

#### Waitlist control app

**Fig. 2:**
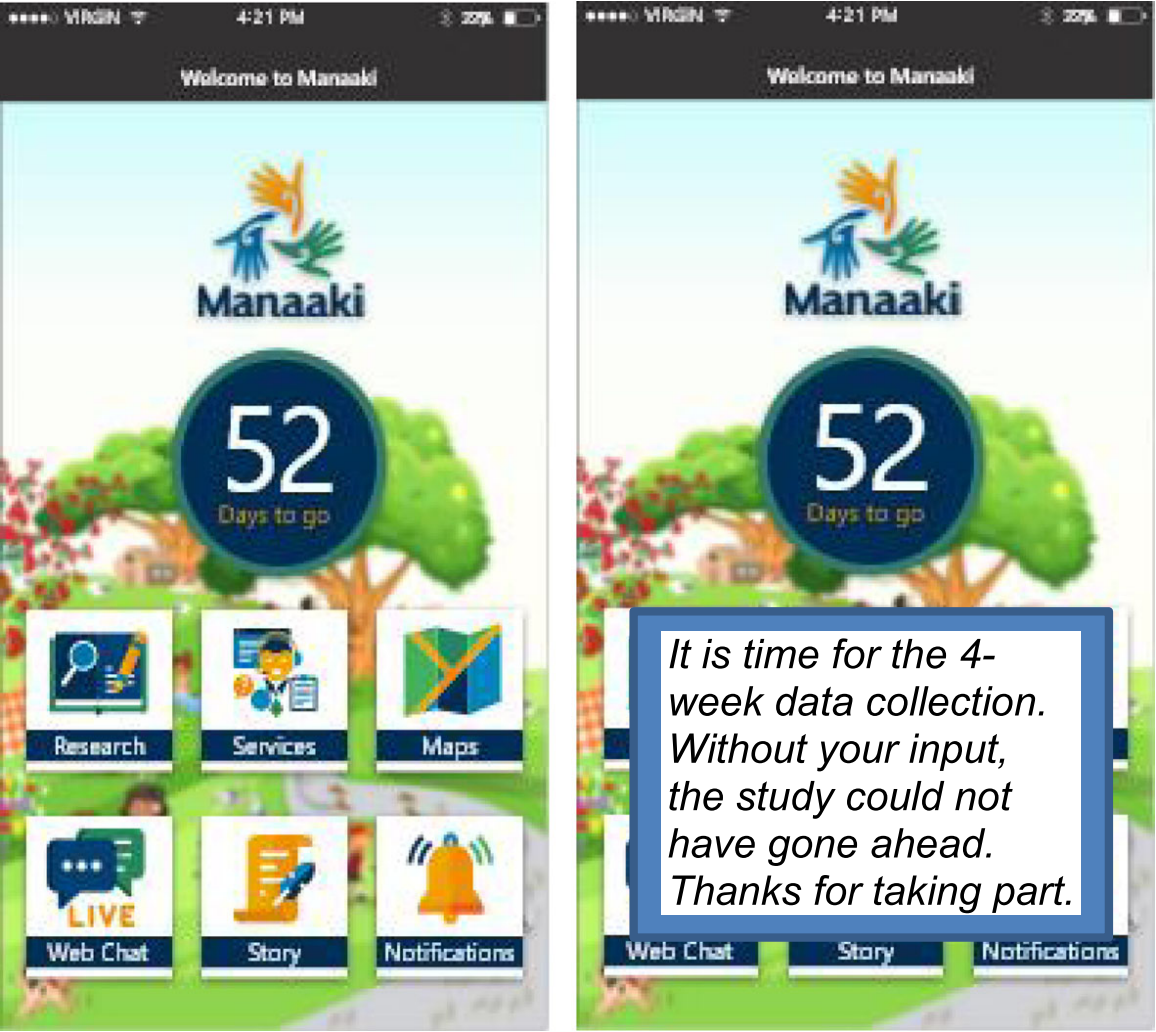
Waitlist Control App Concept Design

Participants randomized to the waitlist control group will have access to the waitlist app which presents a timer that shows participants the days remaining until they are able to activate the full intervention app (Fig. 2), the data collection forms and links to available gambling services. Participants will receive short push notifications (messages) reminding them to complete data collection at 4-, 8-, and 12-weeks and their importance in taking part in the trial. At the end of the 12-week period, upon completion of the final data collection, participants will have full access to the Manaaki app.

### Measures

Table 2 details the schedule of outcome assessments measured at various time points.

**Table 2:** Schedule of baseline and follow-up data collection

Timing	Baseline	4 weeks	8 weeks	12 weeks	Post 12 weeks
Description	Screening, Randomisation, Baseline data collection	Follow-up data collection	Follow-up data collection	Follow-up data collection	Ongoing^b^
General data					
E-informed consent	✓				
Eligibility	✓				
Descriptive Data					
^a^Age, ^a^sex, ^a^ethnicity, Iwi	✓				
Region and sub-region	✓				
Annual Income	✓				
Primary outcome					
^a^G-SAS	✓	✓	✓	✓	✓
Secondary outcome measures					
Frequency, duration & expenditure	✓	✓	✓	✓	✓
Secondary Descriptive Measures					
Problem gambling modes	✓				
Goal setting	✓				
Follow up goal setting		✓	✓	✓	✓
Help-seeking	✓	✓	✓	✓	✓
Readiness to change	✓	✓	✓	✓	✓
App					
App engagement and utilisation, Frequency, Intensity, Time and Type	Continuous throughout the study				*✓*

^a^ Used for randomization

^b^ The app will be available for all participants for at least 12 weeks after the study is completed

#### Primary outcome

The primary outcome is a change in G-SAS at 12 weeks. The G-SAS consists of 12 items designed to assess change in gambling symptom severity during treatment. It uses a past week timeframe with each item scored from 0 to 4, with varying response options for each item. Total scores on the G-SAS range from 0 to 48, with higher scores indicating greater gambling symptom severity. Scores on the G-SAS are categorised as extreme (41–48), severe (31–40), moderate (21–30), or mild (8–20). The G-SAS has demonstrated high internal consistency and good convergent validity with other measures of gambling symptom severity [50].

#### Secondary outcomes

The following secondary outcomes will be assessed.

1. Gambling urges: The first four items of the G-SAS can be used as an assessment of change in gambling urges, with scores ranging from 0 to 16 (α = 0.87).
2. Gambling frequency, time and expenditure: Gambling frequency, time and expenditure will be measured using three questions about the number of days, hours and money spent in the past 4 weeks
3. Readiness to change: Readiness to change which is associated with the trans-theoretical stages of change and participant’s confidence in enabling change, will be assessed by three items. The items are based on readiness, willingness, and how able the participant believes they are capable of making a change. All three are reported using a readiness ruler (0–10 scale) [51]. Readiness rulers have demonstrated good psychometric properties in the measurement of these constructs across other addictions [52, 53].

#### Descriptive measures

1. Demographic data: At baseline assessment, demographic data including age, sex, ethnicity, annual income and geographic location will be collected.
2. Problem gambling activity types: At baseline, a single item will be used to assess all activities participants perceive they have an issue with (i.e. number games; electronic gaming machines; informal private betting for money; table games; horse, harness or greyhound racing; and sports or event betting)
3. Treatment goal: A self-identified treatment goal (quit or reduce time and/or money spent on self-identified gambling mode) will also be collected.
4. Help-Seeking behaviour: Participants will be asked to report on the frequency of help-seeking activities undertaken during the past month using a 0–100 scale measurement ruler [54].
5. App use data: Frequency, Intensity, Time and Type (FITT) will be collected [55] and self-reported engagement and experience questions [36]. This includes 1) the frequency of engagement with the app (i.e. number of different interactions), 2) the intensity of engagement (i.e. Modules viewed, activities completed, actions undertaken), 3) time spent using Manaaki overall, 4) type of app engagement (i.e. active recording of activities and actions, use of active tools versus passive (didactic) information reading, use of assistive tools such as assessments and reflections) and the number of days between each active app use, 5) the pattern of app us (i.e., what modules are accessed and in what order), and 6) self-reported experience attributes such as attractiveness, perspicuity, efficiency, reliability, stimulating, perceived positive effect, depth of use, and attention.

### Sample size calculation

It is hypothesized that exposure to the Manaaki intervention app will result in a 5-point change (reduction) in the baseline score on G-SAS for the participants in the intervention group A 5 point reduction is reported as a significant change in the severity of symptoms [56, 57]. With 90% power, a two-sided alpha at 5%, and an attrition rate of 40%, a sample size of 284 (142 per group) will be required to detect a minimum of a 5-point reduction on the G-SAS. The 40% attrition rate was selected as the worst-case scenario based on gambling intervention attrition rates which ranged from 14 to 50% [58, 59].

### Data analyses

All statistical analyses will be performed using SAS version 9.4 (SAS Institute Inc. Cary NC). Data analyses will be specified a priori in a statistical analysis plan (SAP) prepared by the trial statistician. The data will be imported into SAS for analysis. No interim analyses are planned.

All baseline data will be summarized by treatment group. Continuous outcomes will be analysed using multiple linear regression (ANCOVA) and adjusted for baseline outcome value, the stratification factors used in the randomisation (ethnicity, sex and 31–48) G-SAS below 30 and other covariates if needed. Where there are binary outcomes, simple incidence rates, relative risks, and chi-squared tests will be calculated. Treatment evaluations for the primary outcome will be carried out on an intention-to-treat (ITT) basis, where the ‘last value carried forward’ method will be used to replace missing data. Sensitivity analyses will be conducted to test the robustness of the primary outcome results. These will include per-protocol analysis, complete case analysis, and ITT analyses using multiple imputations to replace missing values. Secondary analyses on the primary outcome will also be conducted using repeated measures mixed models adjusted for baseline outcome value. Similar analyses will be conducted on secondary outcomes using the line function appropriate to continuous or categorical variables. The consistency of effects on the primary outcome will be assessed using tests for heterogeneity for pre-specified subgroups such as ethnicity (Māori, Pacific, Other), sex, age (dichotomised based on the median) and gambling symptom severity groups.

The clinical significance of any effect will be demonstrated by calculating effect sizes presented as Cohen’s d for continuous and normally distributed primary and secondary outcomes. Because Cohen’s d effect sizes are based on the assumption of normality for continuous data, odds ratios (ORs) and CIs will be employed as a measure of effect size for ordinal and categorical outcomes. A clinically significant change, as outlined by Jacobson and Truax [60], will also be evaluated for G-SAS gambling symptom severity. At each evaluation, each participant’s status will be defined as “recovered” (final score falls into the functional range and corresponds to a reliable change), “improved” (final score corresponds to a reliable change, but falls into the dysfunctional range), “unchanged” (final score does not correspond to a reliable change), or “deteriorated” (final score corresponds to a reliable change in the negative direction). On the G-SAS, the functional range is defined as scores falling in the mild range or below (i.e., score of 20 or less).

Missing data will be managed based on the following: 1) follow-up of all randomised individuals will be attempted, using notification prompts through the app and where agreed, via the contact details provided at consent. As the data is captured electronically, the project manager will be able to view who has completed, who has not, and what set of reminder notifications are sent (the research team and statistician will not have access to allocation or any study data), 2) for data collection time points at 4-, 8- and 12-weeks, a two-week timeframe will be allowed for each follow-up assessment. Response intervals and frequency of questionnaire completion will be expected to vary between individuals. The two-week period post due date was selected as reasonable, as all participants will receive a notification 1 week before each data collection time point indicating that they can complete that questionnaire, and 3) if a participant misses a timeframe to contribute to a specific data point, it does not preclude them from completing the next data evaluation point.

## Reporting of results

The CONSORT 2010 statement will be followed as the guidelines for reporting parallel group randomised trials. The overall trial results will be communicated through presentations at national and international conferences, and articles in peer-reviewed scientific journals. Study participants will be informed about the trial results if they select in the consent that they want a copy. The results will be in plain-language and sent by email. The general public will be informed about the trial via posting of the research findings on the University and other relevant websites. Academic papers and summary reports will be provided to the funding body.

Maori will be informed of progress with the trial, and final results will be disseminated via national and regional Māori electronic and print newsletters. Working with our Māori research partner, Hāpai te Hauora, a specific and appropriate message and dissemination strategy will be developed to ensure appropriate dissemination of information to Māori. The use of other Māori media (TV, radio) to disseminate information more widely will also be considered.

The Pacific Island community will be kept informed of the trials progress and final results via reports issued to Pacific Island stakeholder groups. Additionally, with a specific and appropriate message and dissemination strategy to ensure appropriate dissemination of information for Pacific Peoples will also be developed.

## Limitations

This study has potential limitations. First, it is possible that unforeseen technical issues may present barriers to delivering the intervention content. The research team has extensive experience in developing and delivering mhealth interventions and, with an in-house technical team, will work closely to monitor and resolve any technical issues throughout the study period using the appropriate mobile app crash and other technical issue reporting tools. The app will also be pre-tested and have an initial soft launch prior to the full promotion and launch of the full trial. Second, low engagement has commonly been reported in mobile app intervention studies. To ensure that the intervention is appealing and engaging for participants, we developed the app in consultation with people with lived experience of problem gambling and providers of gambling treatment services. Specific attention has been paid to the end-user interface, including graphics, colour, tone, and language. Aspects such as avatars and other strategies for personalizing the app to the user have been incorporated to support app “stickiness” and support participant retention. Thirdly, the presence of the waitlist app used in the control group may have a Hawthorne effect on the findings [61]. To mitigate this, the waitlist app has been designed as a simple placeholder and data collection tool, that has a timer to indicate when the full app will be available. From an ethical viewpoint, it is unethical to completely withhold treatment/intervention for individuals who seek gambling help or support and as such participants in the control group are able to seek any help that they wish during the study period. Finally, the use of a waitlist control group does impact on the ability to measure the effects of the intervention app in the longer term.

Despite these limitations, access and uptake of existing services for gambling problems remain low, and there is a need to explore new approaches to deliver support and treatment for individuals experiencing gambling problems. mHealth offers a promising approach by removing many barriers related to reach and access by high need populations. This has the potential to increase impact at a population level and go some ways to reducing inequality. Information on uptake and adherence to this type of intervention will also be generated to inform future studies for individuals with gambling problems.

## Discussion

This paper presents the design of a pragmatic randomized controlled trial aimed at assessing the effectiveness and acceptability of a mHealth smartphone cognitive behavioural program delivered via a mobile app, for people with self-reported gambling problems. There is currently a lack of evidence-based studies for the use of mHealth tools to support people with gambling problems. Our study will generate knowledge on the impact of the CBT program on the severity of gambling symptoms and other related outcomes and on the engagement, and acceptability on using a mobile app in the gambling domain. Mobile phones have the potential to significantly reduce gambling harm inequalities by reaching vulnerable population groups, regardless of location and other accessibility barriers. If effective, our intervention can be disseminated and delivered widely, rapidly and cost-effectively to population groups that currently report barriers to current intervention modes. Reducing gambling problems has the potential to lead to the wider population benefits for families and communities. The findings will, therefore, be of national and global interest as a new tool for reducing the harms related to gambling.

## Data Availability

All requests for de-identified individual participant data or study documents will be considered, after the publication of the results, where the proposed use aligns with public good purposes, does not conflict with other requests, or planned use by the Study Steering Committee, and the requestor is willing to sign a data access agreement and has sort relevant ethical approvals. Contact will be via the corresponding author.
